# Current diagnostic procedures and interventions for Gaming Disorders: A Systematic Review

**DOI:** 10.3389/fpsyg.2019.00578

**Published:** 2019-03-27

**Authors:** Sebastiano Costa, Daria J. Kuss

**Affiliations:** ^1^Dipartimento di Psicologia, Università degli Studi della Campania Luigi Vanvitelli, Caserta, Italy; ^2^Department of Psychology, Nottingham Trent University, Nottingham, United Kingdom

**Keywords:** gaming disorder, systematic review, clinical studies, clinical procedure, diagnostic criteria

## Abstract

**Background:** Despite the growth in the number of studies on Gaming Disorders (GD), assessing the characteristics of clinical subjects is still limited. Driven by the need to overcome this limitation, a broad systematic review is essential to cover the studies that have already assessed the clinical characteristics of individuals diagnosed with GD.

**Objectives:** The aim of this systematic review is to provide a broad cross-cultural picture of the current diagnostic procedures and interventions used for GDs in clinical practice.

**Methods:** A total of 28 studies met the inclusion criteria, and data were synthesized in these categories: (1) the cultural background of the country where the research took place; (2) the instruments used to measure GD; (3) the diagnostic criteria for GD; (4) the diagnostic procedures used; and (5) the treatment protocol applied.

**Results:** Results of this systematic review suggest that in GD clinical practice, there is a great deal of heterogeneity in the choice of instruments, the diagnostic and intervention processes for GD.

**Conclusions:** This systematic review indicates that a validation process of standard procedures in clinical populations with GD is necessary to create clear shared guidelines for practitioners.

## Introduction

### Rationale

The use of videogames is a rapidly growing phenomenon around the world that involves people of all age groups. The diversity of gaming platforms (e.g., dedicated console, personal computers, smartphones, tablets, and laptops) and the growth in demand have contributed to the gaming industry becoming one of the most profitable entertainment industries (Kuss et al., 2017). The integration with Internet technology has further expanded the use of video games, making the gaming experience even more engaging and immersive. Massively Multiplayer Online Role-Playing Games (MMORPGs) and Multiplayer Online Battle Arena (MOBA) are typical examples of games that combine social interactions in an immersive and challenging environment. Although gaming is a pleasant activity that can also provide interesting educational implications (De Freitas and Griffiths, 2007; Hainey et al., 2016), for a small number of players excessive gaming can result in the development of symptoms traditionally associated with substance-related addictions. Although, gaming is a risk activity only for a small minority of people who tend to play excessively and develop negative symptoms, the public fear about being “addicted to gaming” has been popularized via the media, which in turn spurred the debate on health policy because gaming is a commonly engaged in pastime activity (Billieux et al., 2017; Griffiths et al., 2017). Furthermore, videogames have always been at the center of a public debate with regards to possible health risks they may carry, but the inclusion of *Gaming Disorder* in diagnostic manuals has increased parental and public concerns regarding excessive gaming (Ferguson, 2010).

In the most recent edition of their diagnostic manual for mental disorders, the DSM-5, (American Psychiatric Association, 2013) incorporated *Internet Gaming Disorder* (IGD) in its appendix as a condition that requires further research. In accordance with the DSM-5 definition, the clinical diagnosis of IGD should be characterized by a continuous use of Internet videogames that create significant problems with personal, social, academic, and work functioning. Meeting five of these nine diagnostic criteria within 1 year is indicative of the presence of the disorder: (a) craving, (b) withdrawal, (c) tolerance, (d) relapse, (e) loss of interest, (f) continuance despite problem awareness, (g) deception, (h) mood modification, and (i) jeopardizing work/education/relationships. However, several limitations to these diagnostic criteria have been identified, including the use of the term “Internet” in the terminology of gaming addiction, that exclude the option that gaming addiction could occur both online and offline (Király et al., 2015; Kuss et al., 2017). Following this first step of the American Psychiatric Association, the World Health Organization (WHO) has now decided to include a revised diagnosis of *Gaming Disorder* (GD) in their diagnostic manual, the ICD-11. Several studies recognized that GD is as a global problem associated with several psychological complications (Kuss and Griffiths, 2012). Poor sleep quality, insomnia, decline in work or academic performance, decrease in cognitive ability, difficulties in interpersonal relationships, increase in negative affect, stress, aggression, and hostility, are only some of the serious consequences for the psychophysical health of a person affected by GD (Kuss and Griffiths, 2012).

However, previous studies have repeatedly outlined that the major obstacle in the field that significantly hinders research progress is that the majority of the studies drew their findings from non-clinical and normative community samples (Kuss et al., 2017). Consequently, interest in clinical studies on GD is growing, and several studies have been conducted with clinical populations. However, due to the lack of standardized procedures for clinical populations with GD, decisions about clinical approaches and procedures are made by researchers and practitioners, with the consequence of using heterogeneous approaches and procedures that risk creating more chaos and confusion in an emerging field (Kuss et al., 2017). Furthermore, the lack of clear guidelines and consensus can lead to an overestimation of the problem with the consequence of an increase in false positives, but the opposite risk is not to recognize and not to adequately treat people who need clinical care (Billieux et al., 2017; Griffiths et al., 2017). Accordingly, a systematic review process is fundamental to understanding the clinical practices that are shared between and common to clinicians, and to further investigate processes that can be included in official GD guidelines. Several studies report that adapting existing guidelines can reduce avoidable duplication of efforts caused by the persistent development of new guidelines (Baker and Feder, 1997; Fervers et al., 2006). A number of systematic reviews with clinical studies have been conducted (King and Delfabbro, 2014; Kuss and Lopez-Fernandez, 2016; King et al., 2017; Zajac et al., 2017), but these systematic reviews have focused on the characteristics of those patients diagnosed with GD and/or on the evaluation of training and intervention, without providing information about the clinical processes.

In fact, most of the previous systematic reviews limited the search to studies that included treatment outcomes (King and Delfabbro, 2014; King et al., 2017; Zajac et al., 2017), and therefore did not provide a complete and exhaustive summary of the characteristics of the clinical sample included in, and these studies did not aim to verify the reported treatment results. Examining these studies is relevant to understanding the diagnostic criteria and diagnostic processes that are used to diagnose individuals with gaming addiction. In accordance with recent studies (Király et al., 2015; Kuss et al., 2017), research on GD needs to clarify the diagnostic and clinical processes used in the clinical context. Furthermore, in order to obtain a strong consensus on the diagnostic process of GD, it is essential to identify and deepen the clinical procedures currently in use as reported in the scientific literature. A systematic review is therefore necessary, so that common clinical practices can be identified, while differences and innovations can be studied and deepened. For this reason, also including the clinical studies that did not evaluate treatment outcomes is important to produce the most comprehensive depiction of the procedures currently in use, without omitting important information of the diagnostic process currently used by professionals.

Finally, most of the reviews on gaming addiction with clinically diagnosed individuals focus only on studies that contain quantitative data (King and Delfabbro, 2014; King et al., 2017; Zajac et al., 2017). Although these restrictive inclusion criteria allow reinforcing the methodological approach of understanding GD, it excludes the opportunity to include qualitative studies and case reports that could provide relevant information about the clinical experience of clients with GD. In light of the need to identify a consensus on the diagnostic aspects of GD (i.e., the diagnostic criteria, diagnostic procedure, staff involved, type of treatment, and treatment structure), it is essential to cover the studies that have assessed the characteristics of clinical patients. In the phase of establishing of an official diagnosis, the exclusion of qualitative studies, single cases and case reports could result in creating a gap between research and clinical practice. The present review aims to address this and fill the gap in knowledge by considering the clinical context, diagnostic criteria, diagnostic procedures, practitioners staff involved, as well as the respective treatment protocols applied. Reviewing the instruments, the diagnostic criteria, and the entire diagnostic process (including the staff involved) used in GD patients allows to synthesize current practices for assessment and diagnosis, and can help create a general consensus on GD diagnosis, whilst identifying discrepancies in GD diagnosis. Likewise, a systematic review of the type of treatment and treatment structure can identify the current modalities of intervention for GD to help defining practical guidelines and instructions for practitioners. In addition, this review will examine the cultural background and countries where the clinical studies were conducted. This aspect is relevant because the prevalence rates are particularly diverse across cultures (Kuss et al., 2014) and also because the cultural context can give meanings to the gaming activities based on social norms, shared beliefs, and common practices (Kuss, 2013).

### Objective

In summary, this systematic review aims to provide a broad cross-cultural picture of the current diagnostic procedures and interventions used with GD patients in clinical practice. Accordingly, we reviewed both qualitative and quantitative studies that included patients with gaming disorder, examining the clinical procedures used to diagnose and treat patients with GD, including the cultural background and the country where the studies were conducted, the instruments used to measure GD, the diagnostic criteria, the diagnostic procedure that was used (including the staff involved), and the treatment protocol applied.

## Method

### Protocol, Registration, and Eligibility Criteria

The present systematic review focuses on individuals clinically diagnosed with GD, and is based on qualitative and quantitative studies that describe diagnostic or intervention procedures used in clinical practice. The PRISMA statement for reporting systematic reviews was adopted (Liberati et al., 2009), and the protocol was not previously registered for this review. Inclusion criteria were coded by both authors reaching an agreement regarding the coding process and were: (a) including clinical samples and/or clinical interventions for gaming addiction; (b) containing quantitative and/or qualitative data; (c) being published in a peer-reviewed journal; (d) being available as full text in one of the following languages (spoken languages of the authors): English, German, Polish, and Italian.

### Information Sources and Search Strategy

Existing papers were identified by searching the academic databases Scopus, WoS, PubMed, PsycINFO, and psycARTICLES from February to April 2018. No filter for year of publication was used. Both authors defined a list of agreed English keywords for the systematic search that was grouped in two categories of words (and their derivatives). The first group contained the following words: game^*^ addiction; gaming addiction; game^*^ disorder; gaming disorder; game^*^ dependence; gaming dependence; compuls^*^ game^*^; compuls^*^ gaming; pathologic^*^ game^*^; pathologic^*^ gaming; excessive game^*^; excessive gaming; problematic game^*^; problematic gaming. The second group of words contained the following words: clinic^*^; diagnos^*^; treat^*^; therap^*^; patient^*^; psychotherap^*^; medic^*^; train^*^; counsel^*^; intervent^*^; educ^*^; Psychoeduc^*^.

### Study Selection and Data Collection Process

**Figure 1:**
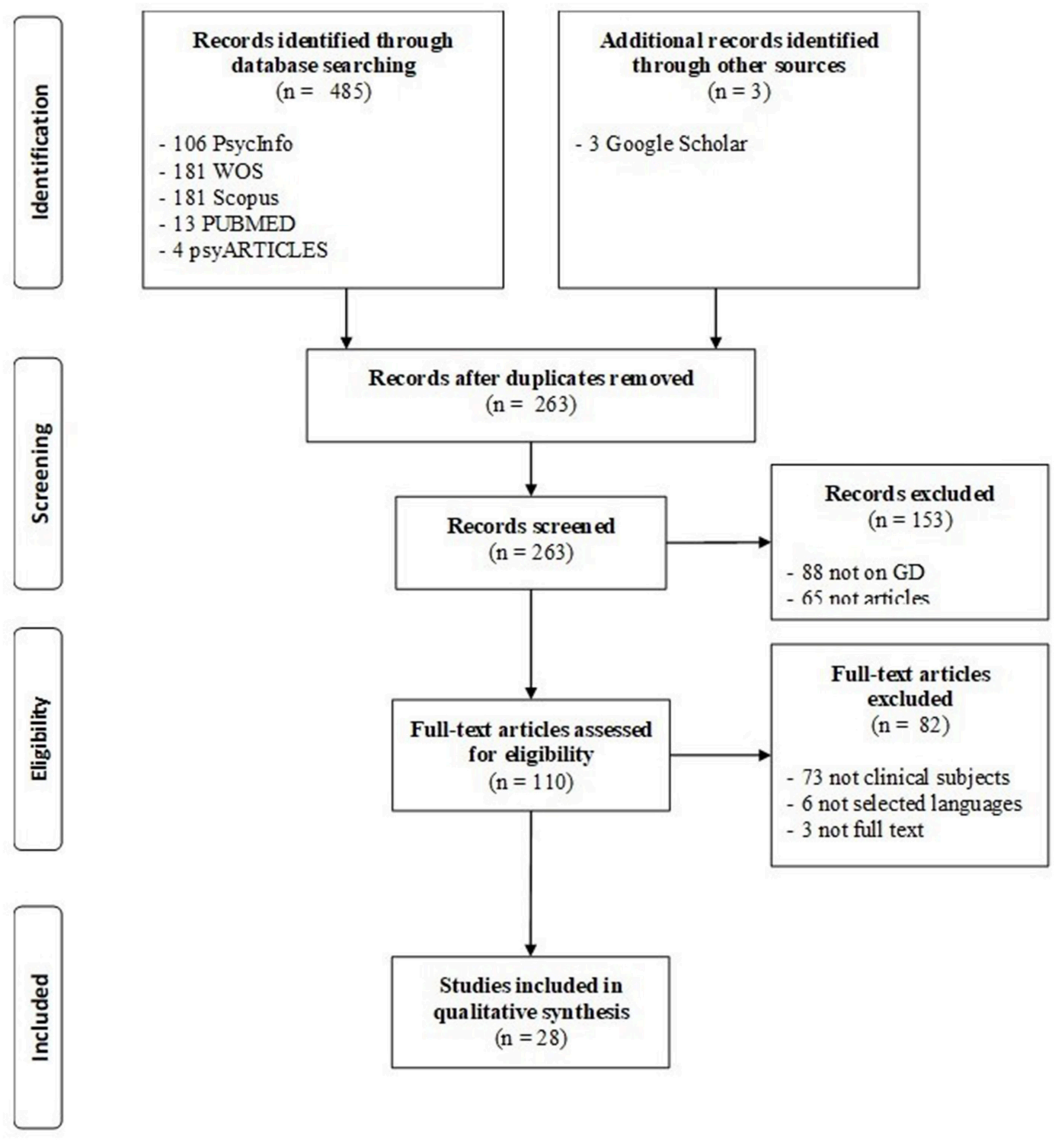
Flow diagram in accordance with PRISMA guidelines (Liberati et al., 2009).

The first search on PsycInfo revealed 106 papers, the second search on WOS found 181 papers, the Scopus search revealed 181 papers, in PUBMED 13 papers were found, and 4 paper derived by the search on psyARTICLES. In a second step, duplicated papers were excluded, and for broad coverage, a search using Google Scholar and reference lists of other papers was conducted, adding three more papers. The selection of papers for the systematic review was based on the inclusion and exclusion criteria previously described. Following the search strategy presented in the flow diagram in Figure 1, the inspection of article titles and abstracts concluded with the inclusion of a total of 28 papers.

### Data Items, Risk of Bias, and Synthesis of Results

Data related to cultural background, the instruments used to measure GD, the diagnostic criteria, the diagnostic procedure conducted, and the treatment protocol applied were obtained from the studies. Considering the exploratory nature of this systematic review and to have a broad understanding of current procedures applied in clinical settings with GD patients, the studies were not filtered according to their quality, and both qualitative and quantitative studies were taken into consideration. Furthermore, a general overview of the risk of bias within studies was evaluated in accordance with the PRISMA guidelines. Each study was evaluated using Cochrane Collaboration's tool (Higgins and Green, 2011) for assessing risk of the following biases: selection bias (describing the quality of allocation to interventions or groups); performance bias (describing the quality of the procedure used during the intervention or evaluation across the groups); detection bias (describing the quality of the procedure in outcomes determination); attrition bias (describing the quality of procedure in the management of missing, withdrawals and incomplete data); reporting bias (describing the quality of procedures in reporting results and outcomes). One or more risks of bias are reported in Supplementary Table 1.

Given the high levels of heterogeneity of the data across studies with regards to research methods, a meta-analysis was not conducted and data were synthesized qualitatively through a summary table and a narrative synthesis using these categories: (1) the cultural background of the country where the research took place; (2) the instruments used to measure GD; (3) the diagnostic criteria for GD; (4) the diagnostic procedures used; and (5) the treatment protocol applied.

## Results

### Study Selection and Characteristics

In this review, a first group of 485 papers were identified by searching for the keyword in the scientific database. As described in the flow diagram, 225 papers were excluded because they were replicated records, 88 papers were excluded because the topic was not GD, 65 records were excluded because they were proceedings abstracts or book reviews (not scientific peer-reviewed papers), 73 papers were excluded because they did not describe clinical patients with GD, 6 papers were excluded because they were written in a language not spoken by the authors, and three papers were excluded because the full-text was not available. A total of 28 studies met the inclusion criteria and these are presented in Supplementary Table 1. The publication dates ranged from 2010 to 2018 and contained clinical samples with a diagnosis of GD.

### Risk of Bias Within Studies

Some studies (Zhang et al., 2016a,b, 2018; Deng et al., 2017; King et al., 2018) were considered at risk for selection bias, because the allocation in the experimental or control group was not random, or because the experimental group was made up only of patients who agreed to participate from a clinical community. In all the studies, it was not possible to estimate performance bias because the blinding procedure of participants and personnel could not be applied because only one of the groups received an intervention (e.g., clinical group vs. healthy group). A risk of detection bias was also reported in one study (Eickhoff et al., 2015) because the outcomes were exclusively based on the reports of the same therapist that conducted the intervention. A number of studies (Eickhoff et al., 2015; van Rooij et al., 2017; Zhang et al., 2016a,b, 2018; King et al., 2018) could have a risk for attrition bias because intervention data were incomplete, or quite large numbers of missing or incomplete data were detected. Some studies (Eickhoff et al., 2015; Park et al., 2016b, 2017; Vasiliu and Vasile, 2017) showed reporting bias because not all the information about outcome and the evaluation of the treatment were reported, or because effect sizes were not reported.

### Synthesis of Results

This review focuses on: (1) the country were the studies were conducted and the examination of the cultural background; (2) the instruments used to measure GD; (3) the diagnostic criteria for GD; (4) the diagnostic procedure that was conducted; and (5) the treatment protocol applied.

### Cultural Background

From the analyses of the included studies, different cultural backgrounds emerged. Although the review is based only on 28 papers, results showed that most of the studies were conducted on the Asian continent, with South Korea being the most frequently represented country with 12 studies. Five studies were conducted in China, two in Taiwan and one in Japan. Five studies were conducted in European countries, and two of these were carried out in Spain, while the remaining single studies were carried out in Germany, in The Netherlands and in Norway. Finally, one study was conducted in the USA and in Australia, respectively. In one study (Vasiliu and Vasile, 2017), the country where the study was conducted has not been explicitly reported. In general, the results show that the largest number of clinical trials had been conducted on the Asian continent, with South Korea being the most representative country. The number of clinical studies in other countries is rather smaller. There appears a large discrepancy of cultural representativeness of GD, showing the need that GD should be further investigated from a cross-cultural perspective.

### Measurement

In the studies included in this review, GD was measured with different instruments. Most of the studies (*n* = 16) used unspecific measures of GD, but general measures of Internet addiction. Eleven studies (Han et al., 2010, 2012a,b; Han and Renshaw, 2012; Kim et al., 2012, 2015; Park et al., 2016a,b, 2017; Lee et al., 2017; Nam et al., 2017) used Young's Internet Addiction Test (IAT; Young, 1996), while six studies used Chen's Internet Addiction Scale (CIAS; Chen et al., 2003). The IAT is a 20-item questionnaire that uses several cut-offs to differentiate Internet users. Nine studies (Han et al., 2010, 2012a,b; Han and Renshaw, 2012; Kim et al., 2012; Park et al., 2016a,b; Lee et al., 2017; Nam et al., 2017) used a cut-off of 50, Kim et al. (2015) used a cut-off of 70, while in the study of Park et al. (2017), the cut-off was not reported. Chen's Internet Addiction Scale (CIAS; Chen et al., 2003) is a 26-item self-report measure that includes five dimensions of Internet use-related symptoms (compulsive use, withdrawal, tolerance, interpersonal relationship problems, and life management). Four studies (Zhang et al., 2016a,b, 2018; Deng et al., 2017) included in this review used the CIAS applying a cut-off of 67 for problematic use, while Ko et al. (2014) and Yao et al. (2017) did not report the cut-off. All the other studies used different measures to assess GD. Müller et al. (2014) used the 13-item self-report scale for the Assessment of Internet and Computer Game Addiction (AICA-S; Wölfling et al., 2011) that derives from the criteria of addiction disorders and allows categorizing GD behaviors into normal (0–6.5 points), moderately addictive (7–13 points), and severely addictive use (≥ 13.5 points). Pallesen et al. (2015) used the Game Addiction Scale for Adolescents (GASA; Lemmens et al., 2009) that consists of 21 items rated on a 5-point Likert scale that refer to seven dimensions of addiction (salience, tolerance, mood modification, withdrawal, relapse, conflict, and problems), and the Problem Video Game Playing Scale (PVGPS; Tejeiro Salguero and Morán, 2002) that consists of nine dichotomous items. The cut-off used by Pallesen et al. (2015) was a score equal to or higher than three on the Game Addiction Scale for Adolescents (GASA; Lemmens et al., 2009). Torres-Rodríguez et al. (2017) used both the Video Game-Related Experiences Questionnaire (CERV; Chamarro Lusar et al., 2014), and the Internet Gaming Disorder Test (IGD-20 Test; Pontes et al., 2014). The Video Game-Related Experiences Questionnaire (CERV; Chamarro Lusar et al., 2014) is a 17-item 4-point Likert scale and used a cut-off equal to or higher than 39, while the Internet Gaming Disorder Test (IGD-20 Test; Pontes et al., 2014) is a 20-item self-report scale on a 5-point Likert scale with a cut-off of higher than or equal to 71. van Rooij et al. (2017) used the Clinical Video game Addiction Test (C-VAT 2.0) and the Video game Addiction Test (VAT; van Rooij et al., 2012). The Clinical Video game Addiction Test (C-VAT 2.0) contains three questions about gaming, and 11 dichotomous questions about past-year GD behaviors based on the 9 DSM-5 criteria for IGD. The Video game Addiction Test (VAT; van Rooij et al., 2012) is a 14-item self-report scale that provides a measure of the severity of the various problematic gaming behaviors (e.g., loss of control, conflict, preoccupation/salience, coping/mood modification, and withdrawal symptoms).

Vasiliu and Vasile (2017) used the Internet Gaming Disorder Scale-Short Form (IGDS-SF; Sarda et al., 2016) that consists of a 9-item self-report based on DSM-5 criteria rated on a 6-point scale ranging from 1 (not at all) to 6 (totally). King et al. (2018) used the Internet Gaming Disorder Checklist (IGD checklist; American Psychiatric Association, 2013) that consists of a 9-item self-report measure rated in a dichotomous way (Yes/No) to assess IGD symptoms in accordance with the DSM-5 IGD classification (preoccupation, tolerance, withdrawal, unsuccessful attempts to limit gaming, deception or lies about gaming, loss of interest in other activities, use despite knowledge of harm, use for escape or relief of negative mood, and harm). King et al. (2018) also included the Internet Gaming Withdrawal Scale (IGWS; Flannery et al., 1999) in their study, which measures frequency and duration of thoughts about gaming, intensity of gaming craving at its strongest point, ability to resist gaming, and overall strength of craving. Finally, three studies (Mallorquí-Bagué et al., 2017; Sakuma et al., 2017; Yeh et al., 2017) used semi-structured clinical interviews with the nine proposed DSM-5 criteria as guidelines and a cut-off of at least 5 criteria or more. Sakuma et al. (2017) also used Griffith's six components of addiction as guidelines for the semi-structured clinical interview (Griffiths, 2005).

Despite this review being based on 28 studies only, overall, a heterogeneous and very diversified use of tools for the evaluation of GD seems to be the norm in the clinical field. Although the differences between the tools used are also attributable to the different time periods in which the studies were written and conducted, the evaluation process has undergone changes (i.e., before and after the publication of the DSM-5), which also indicates that to date, standard and shared criteria for measuring GD have not been identified yet, and no consensus has been reached regarding those. Some tools used for diagnosis are based on the amount of time spent on the Internet, while others are based on the symptoms of the APA's classification of IGD in the DSM-5, or of DSM IV-TR criteria for substance abuse/dependence and pathological gambling. These differences in terms of clinical evaluation impair analyzing and comparing prevalence and incidence rates across studies.

### Diagnostic Process

Different procedures and methods to include subjects in clinical samples were used in the studies. Most of the studies (Han et al., 2010, 2012a,b; Han and Renshaw, 2012; Kim et al., 2012, 2015; Müller et al., 2014; Park et al., 2016a,b; van Rooij et al., 2017; Lee et al., 2017; Mallorquí-Bagué et al., 2017; Sakuma et al., 2017; Torres-Rodríguez et al., 2017) recruited their samples from clinical centers or medical divisions that had previously evaluated patients for GD, and for this reason not much information on the diagnostic process was reported. However, nine of these studies (Han et al., 2010, 2012a,b; Han and Renshaw, 2012; Kim et al., 2012, 2015; Park et al., 2016a,b; Lee et al., 2017) also used a preliminary screening with the Structured Clinical Interview for DSM–IV to evaluate inclusion and exclusion criteria. Two studies (van Rooij et al., 2017; Mallorquí-Bagué et al., 2017) conducted an expansion of any DSM-IV axis diagnoses to complete in accordance with the DSM-V criteria, while four studies (Müller et al., 2014; Park et al., 2017; Sakuma et al., 2017; Torres-Rodríguez et al., 2017) used an external expert rating (performed by psychologists and psychiatrists) to define the inclusion criteria in accordance with the DSM-5.

Six studies (Pallesen et al., 2015; Zhang et al., 2016a,b, 2018; Deng et al., 2017) reported that participants were selected through online questionnaires, newspaper advertisements, and telephone screening, and used in-person semi-structured screening to assess the extent to which the diagnostic criteria were met. However, although the described treatment was conducted by therapists and psychologists, the diagnostic staff and procedures were not described in detail. Similarly, King et al. (2018) screened adults with clinically defined gaming problems who voluntarily visited a website that provided resources to quit or reduce gaming. A psychometric instrument combined with open-ended follow-up questions allowed to check that participants met five or more DSM-5 IGD criteria and personally acknowledged their gaming problems.

Four studies (Han and Renshaw, 2012; Ko et al., 2014; Yao et al., 2017; Yeh et al., 2017) recruited their participants through advertisements, and after a preliminary evaluation of some diagnostic criteria, an interview by a psychiatrist was conducted to determine the diagnosis of IGD. Participants of the study of Nam et al. (2017) were diagnosed after a clinical interview with a psychiatrist. Eickhoff et al. (2015) described the cases of three military personnel that received a diagnosis of GD through military mental health providers, after having experienced several symptoms that interfered with their work activities. The three military personnel were diagnosed individually during the meeting by the military service staff. Vasiliu and Vasile (2017) conducted a psychiatric interview to make a diagnosis. Kim et al. (2013) did not report the diagnostic process that was conducted in their study, but only the diagnostic criteria they used.

Many of the studies included report limited information regarding the entire diagnostic process, and this is a limitation that future clinical studies should overcome. Although the review was based on only 28 studies, what emerges, however, is that most of the studies included an interview conducted by a psychologist or a psychiatrist. Although there is agreement on the use of professional personnel involved in the diagnostic process, the content of the interviews and the levels of structuring sees to vary considerably.

### Diagnostic Criteria

The studies of this review have shown that a combination of criteria is usually used for the diagnosing GD. Most of the studies (Han et al., 2010, 2012a,b; Han and Renshaw, 2012; Kim et al., 2012, 2013, 2015; Ko et al., 2014; Park et al., 2016a,b; Zhang et al., 2016a,b, 2018; Deng et al., 2017; Lee et al., 2017; Nam et al., 2017; Vasiliu and Vasile, 2017; Yao et al., 2017; Yeh et al., 2017) reported game play time as diagnostic criterion, with at least one of these conditions: (a) higher or equal to a range between 2 and 4 h per day; (b) 8 h or more play time per day on weekends; (c) between 14 and 40 h per week. Furthermore, several studies (Han et al., 2010, 2012b; Deng et al., 2017; Yeh et al., 2017) also defined the minimum period of maintaining a pattern of Internet gaming ranging between 1 and 2 years.

Most of the studies also used the DSM-IV criteria for substance abuse (Han et al., 2010; Han and Renshaw, 2012; Kim et al., 2012, 2013, 2015; Park et al., 2016a,b; Lee et al., 2017; Nam et al., 2017), focusing on impaired behaviors or distress due video game play. Ten studies (Ko et al., 2014; Müller et al., 2014; Eickhoff et al., 2015; van Rooij et al., 2017; Deng et al., 2017; Mallorquí-Bagué et al., 2017; Park et al., 2017; Sakuma et al., 2017; Torres-Rodríguez et al., 2017; Yao et al., 2017; Yeh et al., 2017; King et al., 2018) reported that the diagnosis of IGD was established by endorsing at least five or more of the nine DSM- criteria (obsessive use or preoccupation with gaming; withdrawal symptoms; tolerance; failing to stop or reduce gaming; loss of interest in other activities; continued use despite negative consequences; lying to others about the amount of Internet gaming; gaming used to escape or relieve negative emotions; and impairment of interpersonal relationships, job or education).

Some studies also reported the specific IGD symptoms that were evaluated. Eickhoff et al. (2015) reported poor job performance, insomnia, fatigue, poor concentration, irritability and depressed mood as a consequence of gaming. Four studies (Han et al., 2010, 2012b; Han and Renshaw, 2012; Kim et al., 2012) described that the subjects reported a persistent desire for playing Internet games and a failure to reduce gaming. Moreover, a decline in job or academic performance, impairment of interpersonal relationships, disruption of daily routine and diurnal rhythms was reported. Negative emotions and/or oppositional behaviors were also reported when someone asked them to stop playing. Kim et al. (2013) reported that patients tended to report dramatic drops in academic status, social phobic and/or lethargic behavior. Torres-Rodríguez et al. (2017) reported four addictive cases who became irritable when they could not play, who had given up their hobbies, ceased to interact with friends, increased conflicts at home, had a decline in their academic performance, craving to play video games, psychological dependence, and an inability to control their behaviors. van Rooij et al. (2017) reported that participants spent all their free time and even part of their school time on gaming. Moreover, the majority of patients had problems with family and wider social circles were disrupted and school performance declined. Vasiliu and Vasile (2017) reported a case study of a patient that gradually increase the daily hours spent on gaming activities with negative academic consequences, a sense of losing control over his gaming-related activities, neglected his duties around the house and his social relations (separating from his girlfriend, and losing the majority of his non-gaming friends).

The criterion that the included studies pay most attention to is that of clinically significant impairment (i.e., jeopardizing work/education/relationships, impaired behaviors). This may be due to the fact that requests for professional support emerge when the gaming experience leads to significant repercussions in everyday life. Other than this specific criterion, the studies included in this review use different criteria for diagnosing GD. Some studies have used DSM IV-TR criteria for substance abuse, others the IGD criteria in the DSM-5, and several studies based the diagnosis mainly on the amount of time dedicated to gaming. Naturally this discrepancy may also be attributed to the different time periods in which the articles were conducted and published. Although in this review it was not possible to assess the relationship between publication times and the diagnosis options, nine papers were published after 2015 (Kim et al., 2015; Park et al., 2016a,b; Zhang et al., 2016a,b, 2018; Deng et al., 2017; Lee et al., 2017; Nam et al., 2017), and reported internet use and/or scores on general Internet addiction tools as diagnostic criteria. Studies on clinical patients take a long time and it is therefore normal that changes take place when the study has already been conducted or started. Unfortunately, only few studies indicate which of the diagnostic criteria the clinical subjects have to fulfill in order to make a diagnosis, impairing to identify the validity and reliability of the diagnostic criteria for GD.

### Treatment

Eighteen studies conducted a treatment for GD, and most of these (Han et al., 2010; Han and Renshaw, 2012; Kim et al., 2012, 2013; Eickhoff et al., 2015; Pallesen et al., 2015; Nam et al., 2017; Park et al., 2017; Torres-Rodríguez et al., 2017; Vasiliu and Vasile, 2017) used an individual approach, which was applied to outpatients, apart from seven studies (Park et al., 2016b; Zhang et al., 2016a,b, 2018; Deng et al., 2017; Sakuma et al., 2017; Yao et al., 2017) that used group therapy approaches, and (Han et al., 2012a) used family therapy.

Individual therapies varied in approach and several aspects, using psycho-educative training, sleep hygiene, and virtual reality therapy (Kim et al., 2013; Eickhoff et al., 2015; Park et al., 2016b; Torres-Rodríguez et al., 2017). Generally the most frequently used approach was Cognitive Behavioral Therapy (CBT) for individual therapy (Kim et al., 2012; Pallesen et al., 2015; Torres-Rodríguez et al., 2017; Vasiliu and Vasile, 2017), and Yao et al. (2017) and Park et al. (2016b) used a group behavioral intervention. CBT ranged typically from 8 to 10 sessions, and each session lasted between 1 and 2 h. Craving Behavior Intervention (CBI) was the most used group treatment that consisted of 2.5–3 h of several topic sessions organized in: (1) warming up exercise, (2) a discussion about the homework from the last session, (3) a main structured activity, (4) a brief summary, (5) and the homework assignment. Five studies used a pharmacotherapy intervention. These were mostly based on bupropion sustained release (SR) treatment (Han et al., 2010; Han and Renshaw, 2012; Kim et al., 2012; Nam et al., 2017), while Park et al. (2017) used pharmacotherapy with a selective serotonin reuptake inhibitor.

All 18 studies which used a treatment reported the reduction of symptoms of GD and/or the frequency of gaming to verify treatment effectiveness. Of these, six studies (Han and Renshaw, 2012; Kim et al., 2012; Nam et al., 2017; Sakuma et al., 2017; Yao et al., 2017; Yeh et al., 2017) also examined psychological health indicators such as depression, impulsivity, anxiety, self-esteem, and life satisfaction. Furthermore, in five studies (Han et al., 2010; Park et al., 2016b; Zhang et al., 2016a,b, 2018) neuropsychological changes were assessed via fMRI. Han et al. (2012a) also showed an improvement in perceived family cohesion, while Kim et al. (2013) showed an improvement in writing and speaking ability. Generally, all the reviewed studies suggest that interventions lead to improvements in patients with GD, underlining how there is a need for interventions to help with the problematic experience related to dysfunctional use of gaming (Griffiths et al., 2017).

Taken together, the results show that clinical studies primarily use CBT interventions and psycho-pharmacotherapy. However, the inclusion of case studies and clinical reports has highlighted how other types of interventions, such as psycho-educative training, sleep hygiene and virtual reality therapy, are currently used in clinical practice. This suggests it may be useful to also examine the frequently used therapeutic practices to create and validate reliable and effective guidelines.

## Discussion

Since the release of the DSM-5 diagnostic criteria for IGD in 2013, it has emerged that the lack of clinical studies is one of the major limitations for comprehensively understanding the phenomenon of GD (Griffiths et al., 2016). For this reason, the objective of this review was to identify and schematize the results of the studies that have used subjects diagnosed with GD. To try developing a complete picture of the clinical practices currently used in various countries, it was necessary to include both qualitative and quantitative studies that have used GD clinical samples both online and offline, which were not limited to studies that included treatment outcomes. The results of this research led to the identification of 28 studies that were deepened and categorized based on: (a) the cultural background where the studies were conducted; (b) measures of GD; (c) the diagnostic criteria for the diagnosis; (d) the diagnostic procedure applied; and (e) the eventual treatment protocol applied.

### Summary of Evidence

In terms of cultural background, results have clearly shown that most of the studies were conducted in the Asian continent (20 studies on a total of 28), with more than half conducted in South Korea. This confirms previous considerations using clinical samples (Király et al., 2015; Kuss and Lopez-Fernandez, 2016) that reported the progress of the policies on the technological addiction in South Korea, which has established a center and large-scale projects to deal with the issue starting in 2002, while in the US and in most European countries GD treatment is not even covered by national health funding.

Regarding the instruments used to assess GD, the most contradictory aspect that is highlighted is that despite a review (King et al., 2013) identified 18 specific tools for the evaluation of GD symptomatology, most studies with clinical patients tend to use general Internet addiction tools, such as the IAT (Young, 1996), and the CIAS (Chen et al., 2003). Naturally this discrepancy is due to the fact that studies on clinical patients need a long time to be concluded and published, and for this reason most of the studies now available are based on measures and criteria which may now appear obsolete, but which were common and widely used in the planning and beginning stages of the studies. In fact, the use of general internet addiction tools may be the result of the tendency to consider GD as a subdomain of Internet addiction. This vision, which derives from a very early conceptualization of GD (Pontes and Griffiths, 2014), has been amplified by the DSM-5 use of the “Internet” terms in the definition of GD criteria. Despite this paradox, it is evident how the studies using GD specific tools in the diagnostic process are increasing, including the following tools: AICA-S (Wölfling et al., 2011), GASA (Lemmens et al., 2009), PVGPS (Tejeiro Salguero and Morán, 2002), CERV (Chamarro Lusar et al., 2014), IGD-20 Test (Pontes et al., 2014), VAT (van Rooij et al., 2012), and IGDS-SF (Sarda et al., 2016). However, the risk remains that such a large and varied number of instruments does not help defining and validating a universal standard for assessment. A step forward regarding the question of measurement was provided by van Rooij et al. (2017), who performed a clinical validation of the C-VAT 2.0, using a clinical sample to test the sensitivity and improve the correct identification of patients diagnosed with GD. Although all the above mentioned instruments showed good psychometric properties, such as reliability and construct validity, only the C-VAT 2.0 reported good clinical validity.

In terms of the diagnostic process, it is difficult to obtain a complete picture of how the subjects were diagnosed, since most of the patients were independently diagnosed in clinical centers and then recruited for study purposes, rather than being followed by the research team through the whole diagnostic process. Despite this limitation, several interesting conclusions on the procedure used in these clinical studies can be drawn. A characteristic present in most of the studies is that an interview (with different levels of structuring) with a professional such as a psychologist or a psychiatrist or the evaluation of multidisciplinary staff is beneficial for diagnosis. In many studies, such an interview had the function of evaluating the criteria for inclusion and exclusion of participants for the respective research, and it can be considered a good practice for a complete evaluation (American Psychiatric Association, 2013). One aspect to keep in mind is also the fact that many studies have often used the diagnostic criteria of DSM-IV-R in their evaluations and only in some cases have they been updated to the DSM-5 criteria. This anomaly derives from the patients included in the studies having been diagnosed within the intervention centers before the DSM-5 criteria were released, and in some cases, the research on clinical samples was published before the publication of the new diagnostic manual. This aspect further emphasizes that it is necessary to conduct more frequent examinations on clinical samples to allow consolidation of the new diagnostic criteria and establish a unique standard for assessment and diagnosis of GD (Kuss and Lopez-Fernandez, 2016; Kuss et al., 2017).

Connected to the previous point, the DSM IV-TR criteria for substance abuse/dependence were often used to define GD (tolerance, withdrawal, intended effects, loss of control, excessive time spent gaming, continuity despite problems, and reductions of other activities). There are still fewer studies (ten in the present review) that used the DSM-5 criteria to define the diagnosis instead: (a) craving, (b) withdrawal, (c) tolerance, (d) relapse, (e) loss of interest, (f) continuance despite problems, (g) deception, (h) mood modification, and (i) jeopardizing work/education/relationships. In addition to the DSM criteria, most of the studies have also used the presence of impaired behaviors or distress because of videogames as diagnostic factors and a high frequency of videogame use with these specific cut-offs: (a) greater or equal to a range between 2 and 4 h per day; (b) 8 h or more play time per day on weekends; and (c) between 14 and 40 h of gaming per week. Some case studies also showed patients diagnosed with GD experienced the following symptoms: deterioration of school or work performance, negative mood, impairment of interpersonal relationships, given up hobbies, disruption of daily routine and diurnal rhythms, negative emotions, or oppositional behaviors as a result of the request to stop playing, and sense of losing control over gaming-related activities. Finally, the studies presented often cited withdrawal and tolerance symptoms, for which there is no total scientific agreement to date (Király et al., 2015). Accordingly, publication times are important to consider. Most of the studies which are described, in fact, have been defined and conducted when the DSM-5 criteria were not yet available. It is reasonable to expect that diagnosis and treatment of GD will evolve over time, becoming more accurate and effective as time passes. However, if at this time it is possible to observe a considerable heterogeneity of procedures which have been used in the past, with the release of the DSM-5 and the ICD-11, there may be a risk that in the future there will be even greater fragmentation over time.

It is evident that in clinical practice, the guidelines of the DSM are always guiding the diagnosis and treatment. If in the past GD diagnosis was guided by the adaptation of the criteria for substance dependence, the inclusion of IGD in the DSM-5 has certainly provided an important first step to share specific criteria. However, from the analysis of the results, it seems clear that the diagnostic criteria need to be validated in clinical settings. At present, it is not possible to clearly demarcate pathological behaviors from non-pathological ones. A clear example can be represented by the criterion of the amount of time spent playing. Although most of the studies in this review used this criterion for the diagnosis of GD, previous studies showed that professional gamers need to spend considerable amounts of time gaming (Faust et al., 2013), but this does not imply that they must necessarily develop addiction (Kuss et al., 2012). In this review, it was not possible to analyze information about false positives and false negatives which psychologists and psychiatrists are confronted with in the context of diagnosis. However, future studies should deepen this process because it is of fundamental importance to identify sensitive and specific diagnostic processes, and accurate cut-off points. Another aspect to take into consideration is the reliability of diagnostic criteria with respect to time and contexts. To have an effective diagnostic process it is also necessary that the diagnoses are reliable and similar if repeated after short periods of time, or when repeated by different individuals (e.g., different professional staff) or in different contexts (e.g., different clinics). At present, the heterogeneity of instruments, diagnostic criteria and cut-offs makes the diagnostic procedure unclear. Accordingly, there is a strong need for studies aimed at validating the criteria of GD.

Finally, the last aspect described in this systematic review is treatment procedure. Results confirm previous reviews on treatment studies in IA and GD (King and Delfabbro, 2014; Kuss and Lopez-Fernandez, 2016; King et al., 2017; Zajac et al., 2017), suggesting that the most commonly applied therapy forms are CBT (and its variations) and psychopharmacotherapy. A relevant aspect that emerged from this systematic review is that in clinical practice could also use different approaches that are usually described in research reports. Some studies (Kim et al., 2013; Eickhoff et al., 2015; Park et al., 2016b) used as individual or group therapy: psycho-educative training, sleep hygiene, and virtual reality therapy. Sleep hygiene was found to be a procedure that helped in symptom management of GD because extended night-time gaming was found to jeopardize work performance and health. Sleep hygiene consists of different practices to help patients obtaining good sleep habits. Moreover, Virtual Reality Therapy (VRT) is a psychotherapy method that uses virtual reality technology and consists of three steps of relaxation, simulation of a high-risk situation, and sound-assisted cognitive restructuring, leading important reductions in GD severity. Finally, Kim et al. (2013) reported how students with GD benefitted from education training in writing and speaking using narrative aspects borrowed from gaming. These types of interventions generally are not presented in previous systematic reviews and are not normally described as typical training techniques for GD. For this reason, it is necessary to make a call aimed at a greater dissemination of all the interventions carried out by clinicians around the world with GD patients. This would allow to amplify its diffusion and verify its effectiveness through new reviews and meta-analyses. In a new field like that of GD where the diagnostic and therapeutic process is still in progress, the risk of traveling between two parallel paths between those who already work in the clinic on a daily basis and who verify the effectiveness of the treatments and conduct research must be avoided.

### Limitations

Results of this systematic review should be considered in light of the included studies' limitations. The first limitation is that unpublished material has not been included. This could create a publication bias with regards to the tendency to publish positive results more frequently. Another relevant limitation is that only papers published in some languages were added in the review. This could exclude some relevant papers written in non-English countries which reported results in their native language. Furthermore, this systematic review only has a descriptive and explorative purpose and could not verify the quality of the diagnostic processes and that of treatment. Future studies should try to conduct a more specific evaluation of clinical studies, and should focus more on clinical aspects of GD to establish clear and shared guidelines for practitioners.

### Implications and Conclusions

From the review of the clinical studies, there is a great deal of heterogeneity in the choice of instruments, in the diagnostic and intervention processes. If the publication of the DSM-5 criteria was an “earthquake” for the GD field (Kuss et al., 2017), it is likely that the publication of the IGD-11 will have similar effects. For this reason, it is necessary to create a common basis for researchers that can guide clinical practice and that allows for collaboration and growth in the field. The validation of standard procedures in clinical populations with GD seems to be a priority necessary for future research. Regarding political implications, there is a need to stipulate protocols of collaboration with national and international boards to establish treatment and prevention centers all over the world in order to speed up the standardization process of the guidelines for the management of patients with GD.

## Author Contributions

SC generated the initial draft of the manuscript, conducted the identification and the search of the papers to include in the systematic review. DK supervised and coordinated the entire work, prepared, wrote, and edited the manuscript.

### Conflict of Interest Statement

The authors declare that the research was conducted in the absence of any commercial or financial relationships that could be construed as a potential conflict of interest.

## Supplementary Material

Table S1 Summary of studies reviewed.
