# Supplementary material for: Current diagnostic procedures and interventions for Gaming Disorders: A Systematic Review
Source: Front Psychol. 2019 Mar 27;10:578. doi: 10.3389/fpsyg.2019.00578 (PMC6445881; doi:10.3389/fpsyg.2019.00578)
Supplement: Table S1 — Summary of studies reviewed. [file Table_1.pdf]

	Paper	Country	Aims	Sample	Measures	Gaming Addiction Diagnostic Criteria	Diagnostic Procedure	Treatment	Results	Possible Risk of Bias
1.	Deng et al., (2017)	China	Test the effectiveness and detect the active ingredients of craving behaviour intervention (CBI) in mitigation of IGD among young adults.	63 male college students with IGD were assigned into the intervention group (44 participants) or the waiting-list control group (19 participants).	Chen Internet Addiction Scale (CIAS; Chen et al., 2003)<br>Fagerstrom Test for Nicotine Dependence (FTND; Fagerstrum, 1978)<br>Alcohol Use Disorder Identification Test (AUDIT-C; Bush et al., 1998)<br>Michigan Alcoholism Screening Test (MAST; Selzer, 1971)<br>Beck Depression Inventory II (BDI-II; Beck et al., 1996)<br>Beck Anxiety Inventory (BAI; Beck et al., 1988)	Inclusion criteria:<br>(1) CIAS ≥ 67;<br>(2) Internet gaming > 20h/week for minimum 1 year;<br>(3) Internet gaming as primary online activity.<br>Exclusion criteria:<br>(1) DSM-5 criteria (through a semi-structured interview) for abuse or dependence of substances, including alcohol;<br>(2) current or history of use of illegal substances and any gambling experience (including online gambling).	Internet Gaming Addiction participants selected through an online questionnaire and telephone.<br>Participants reported the hours they spent on Internet using.<br>Diagnostics Staff not reported, but 4 therapists with a clinical background in behavioural therapy and group therapy conducted the interventions.	Craving Behaviour Intervention (CBI) face-to-face group therapy program, once a week for 6 weeks.	Compared to the control group, a significant decrease in the severity of IGD in intervention group was found at post intervention and lasting to 6 months after intervention.	Selection Bias
2.	Eickhoff et al., (2015)	U.S.A.	Report three case of military members where poor job performance, insomnia, and depression were associated with<br>sleep deprivation because of 30 to 60 hours of online video<br>gaming per week.	3 military members evaluated by mental health providers because of poor job performance, insomnia, and depressed mood.	N/R	IGD symptoms based on DSM-5 criteria:<br>(1) withdrawal symptoms;<br>(2) continued excessive use of Internet games<br>(3) use of Internet games to escape or relieve negative mood<br>(4) work performance jeopardized by Internet gaming, and unsuccessful<br>attempts to cut back<br><br>video gaming: between 30h and 60h per week.	3 cases of military service members evaluated by mental health providers because of poor job performance, insomnia, and depressed<br>mood.<br>Symptoms were associated with sleep deprivation;<br>Drowsiness, fatigue, poor concentration, irritability, poor work performance, and blunted affect.<br>Patients also exhibited craving, anger, and irritability when not able to play video games.<br>Staff: Military mental health providers	Individual Therapy; Education on sleep hygiene;<br>Forced Video Gaming reduction<br><br>Patient 1: The patient was given education on sleep hygiene to include shutting off the computer 30 minutes to an hour before sleep<br>Patient 2: The patient was educated on sleep hygiene and did not have access to video gaming.<br>Patient 3: The patient was provided education on sleep hygiene, alcohol abuse, and Internet Game Disorder	Reporting video gaming addiction in patients presenting with sleep disturbances, poor work performance, and depressive symptoms	Detection Bias;<br>Attrition Bias;<br>Reporting Bias
3.	Han, Hwang, & Renshaw (2010)	South Korea	Bupropion sustained release (SR) treatment would decrease craving for Internet game play as<br>well as video game cue-induced brain activity in patients with Internet video game addiction<br>(IAG).	Eleven subjects who met criteria for IAG, and eight<br>healthy comparison subjects who had experience playing StarCraft.	Young Internet Addiction Scale (IAT; Young, 1996);<br>Beck Depression Inventory II (BDI-II; Beck et al., 1996)<br>Structured Clinical Interview for DSM–IV (APA, 2000)	game play time > 4 hr per day and 30 hr per week);<br>(1) score ≥ 50 on the IAT<br>(2) impaired behaviours or distress because of videogames consistent with DSM–IV criteria for substance abuse.	Only subjects with inclusion criteria to<br>the specific game "StarCraft", previous evaluated by the Department of Psychiatry.<br>Diagnostic Evaluation:<br>Increasing time of gaming.<br>-Persistent desire to play internet games;<br>-Difficulty in reducing or controlling online game play;<br>-Decline in academic or work performance;<br>-Disrupted diurnal rhythms;<br>-Negative feelings when stop gaming.<br>-Life problems because of gaming	Bupropion<br>To take bupropion SR (Wellbutrin SR, Glaxo Wellcome Inc.) daily for 6 weeks. Bupropion SR was started at 150 mg/day during the first week and then increased to 300 mg/day thereafter. SR	After a 6 week period of bupropion SR, craving for Internet video game play, total game play time, and cue-induced brain activity in dorsolateral prefrontal cortex were decreased in the IAG.	-
4.	Han, Lyoo, & Renshaw, 2012	South Korea	Brain regions consisting of anterior cingulate, thalamus and occpito-temporal areas may increase the likelihood	Twenty patients with on-line game addiction, seventeen	Young Internet Addiction Scale (IAT; Young, 1996);	Inclusion Criteria<br>(1) game play time > 4h per day and > 30h per week;	Subjects previous evaluated by the Department of Psychiatry using inclusion criteria.	N/A	Compared to HC, the patients with on-line game addiction group showed increased impulsiveness and perseverative	-

			of becoming a pro-gamer or patients with on-line game addiction.	pro-gamers, and eighteen healthy Comparison.	Beck Depression Inventory II (BDI-II; Beck et al., 1996)<br>Structured Clinical Interview for DSM–IV (APA, 2000)	(2) score > 50 on the IAT<br>(3) impaired behaviours or distress due video game play;	Increasing time of gaming.<br>-Persistent desire to play internet games;<br>-Difficulty in reducing or controlling online game play;<br>-Decline in academic or work performance;<br>-Disrupted diurnal rhythms;<br>-Negative feelings when stop gaming.<br>-Life problems because of gaming		errors, and volume in left thalamus gray matter, but decreased gray matter volume in both inferior temporal gyri, right middle occipital gyrus, and left inferior occipital gyrus, compared with HC	
5.	Han & Renshaw, (2012)	South Korea	Bupropion treatment would reduce the severity of excessive online game play as well as depressive symptoms.	Fifty male subjects with comorbid EOP and MDD were randomly assigned to bupropion and education for internet use (EDU) or placebo and EDU groups.	Young Internet Addiction Scale (IAT; Young, 1996);<br>Beck Depression Inventory II (BDI-II; Beck et al., 1996)<br>Structured Clinical Interview for DSM–IV (APA, 2000)	Inclusion Criteria<br>(1) game play time > 4h per day and > 30h per week;<br>(2) score > 50 on the IAT<br>(3) impaired behaviours or distress due video game play;	Participants were screened with the Structured Clinical Interview for DSM-IV and diagnosed by a psychiatrist.<br><br>Diagnosis based on inclusion criteria to be consistent with DSM-IV criteria for substance abuse.<br>-Increasing time of gaming.<br>-Persistent desire to play internet games;<br>-Difficulty in reducing or controlling online game play;<br>-Decline in academic or work performance;<br>-Disrupted diurnal rhythms;<br>-Negative feelings when stop gaming.<br>-Life problems because of gaming	Bupropion and education for internet use or placebo and education for internet use<br><br>Bupropion SR 150 mg/day and increased to 300 mg/day during the first week of treatment. During weeks 2–7 maintain a consistent dose of bupropion SR. Subjects received drug bottles containing bupropion SR (150 mg/300 mg) on the first day of each weekly education session during the eight-week medication trial period.<br><br>Subjects received once a week of eight education sessions focused on healthy internet use and the adverse consequences of excessive video game play.	Addiction scores, the mean time of online game playing, and depression in the bupropion group were reduced compared with those of the placebo group,	-
6.	Han, Kim, Lee, & Renshaw (2012)	South Korea	Evaluated the efficacy of a brief 3-week family therapy intervention in the change patterns of brain activation in response to affection and gaming cues in adolescents from dysfunctional families who met criteria for on-line game addiction.	Fifteen adolescents with on-line game addiction and fifteen adolescents without problematic on-line game play and an intact family structure.	Partnership, Growth, Affection, and Resolve (FAPGAR; Smilkstein, 1980);<br>Family Adaptability and Cohesion Evaluation scale (FACES III; Olson, 1986);<br>Young Internet Addiction Scale (IAT; Young, 1996);<br>Beck Depression Inventory II (BDI-II; Beck et al., 1996)<br>Structured Clinical Interview for DSM–IV (APA, 2000)	Inclusion Criteria<br>(1) game play time > 4h per day and > 30h per week;<br>(2) score > 50 on the IAT<br>(3) impaired behaviours or distress due video game play (which are modified from DSM-IV criteria for substance abuse);	From adolescents and their parents who visited the Department of Psychiatry of Chung Ang University Medical Center for evaluation and treatment of possible on line game addiction.<br><br>Using inclusion criteria for diagnosis.<br><br>At the first visit and the final evaluation visit, the families visited Chung Ang University Hospital for assessment of brain activity and the severity of problematic on-line game play by the amount of on-line game play and Young Internet Addiction Scale (IAT) scores.<br>Family Therapy was conducted by a Psychiatrist.	Family therapy over 3 weeks focused on family cohesion, and improvement in family dynamics.	Improvement in perceived family cohesion following 3 weeks of treatment was associated with an increase in the activity of the caudate nucleus in response to affection stimuli and was inversely correlated with changes in on-line game playing time.	-
7.	Kim, Han, Lee, & Renshaw (2012)	South Korea	evaluate the efficacy of cognitive behavioural therapy (CBT) in combination with bupropion	Sixty-five depressed adolescents with excessive on-line game play were	Young Internet Addiction Scale (IAT, Young, 1996).	Inclusion Criteria<br>(1) game play time ≥ 4h per day and > 30h per week;	In the Center for Internet Addiction at Chung Ang University Hospital, subjects with problematic online game play and	Bupropion; Cognitive behavioural therapy (CBT)	CBT in combination with bupropion may be effective for the treatment of depressed adolescents with on-line game	-

			for treatment of problematic on-line game play in adolescents with comorbid major depressive disorder.	randomly assigned to a CBT group (N = 32) or a clinical control group (N = 33).	Beck Depression Inventory II (BDI-II; Beck et al., 1996)<br>Beck Anxiety Inventory (BAI, Beck, Epstein, Brown, & Steer, 1988).<br>School Problematic Behaviour Scale (SPBS; Baker & Siryk, 1984).<br>Student's Life Satisfaction scale (Huebner, 1991).	(2) score > 50 on the IAT<br>(3) impaired behaviours or distress due video game play;	major depressive disorder agreed to participate in a randomized trial of CBT.<br>The definition of problematic on-line game play was based on the inclusion criteria.<br>These criteria for on-line game addiction were based on DSM-IV criteria for substance abuse.<br>-Increasing time of gaming.<br>-Persistent desire to play internet games;<br>-Difficulty in reducing or controlling online game play;<br>-Decline in academic or work performance;<br>-Disrupted diurnal rhythms;<br>-Negative feelings when stop gaming.<br>-Life problems because of gaming	All participants were prescribed bupropion with a fixed schedule of 150 mg/day for 1 week followed by 300 mg/day for 7 weeks.<br><br>Participants also received an eight-session Cognitive behavioural therapy (CBT) once a week.<br><br>The CBT is provided by a multidisciplinary treatment team including a psychiatrist, nurse, psychologist, and social worker.<br>Eight session lasts 1.5–2 h.<br>(1) Introduction and the review of adverse consequences of on-line game addiction;<br>(2) Motive for on-line game play and irrational belief systems pertinent to on-line game addiction;<br>(3) Problem solving;<br>(4) Decision making,;<br>(5) Communication skills;<br>(6) Selfcontrol skills;<br>(7) Family therapy;<br>(8) Planning for the future.	addiction, particularly in reducing on-line game play and anxiety, as well as in improving life satisfaction.	
8.	Kim, Kim, Shim, Im, & Shon, (2013)	South Korea	Investigate the use of "Dungeon & Fighter" as an educational tool in a writing and speaking course and examined the MMORPG's educational value for students addicted to the game.	A total of 59 male high school students who were addicted to online<br>Gaming. 27 in the experimental group and 32 in the control group.	EEG recording;<br>KBS speech performance and writing test;	Playing the game every day for time ≥ 4 h per day<br><br>social phobic and/or lethargic behaviour; dramatic drop in academic status;	Addiction to DF was defined as playing the game every day for at least 4 h per day.<br>- Social phobic and/or lethargic behaviour.<br>- Drop in academic status.<br>-Increasing time of gaming.	Writing and speaking course designed by the Korean Broadcasting System (KBS).<br><br>The writing and speaking course were conducted on a total of 21 occasions;<br>Testing required 2 h per day and was conducted over a period of 2 months.	Participants in the experimental group improved their writing and speaking ability far more than<br>those in the control group.	-
9.	Kim et al., (2015)	South Korea	Identify the neurobiological features of the resting-state brain of patients with Internet Gaming Disorder, alcohol use disorder, and healthy controls	45 young males; 16 were diagnosed with Internet Gaming Disorder, 14 with alcohol use disorder, and 15 healthy controls.	Young's Internet addiction test (IAT; Young, 1996);<br>Beck Depression Inventory II (BDI-II; Beck et al., 1996)<br>Beck Anxiety Inventory (BAI; Beck et al., 1988);<br>Barratt Impulsiveness Scale-11 (BIS-11; Barratt, 1985)<br>Structured Clinical Interview for DSM–IV (APA, 2000)	Inclusion Criteria<br>(1) game play time ≥ 4h per day and > 30h per week;<br>(2) score > 70 on the IAT	All patients were<br>seeking treatment at the outpatient clinics of SMG-SNU Boramae Medical Center in Seoul, South Korea due to excessive participation in Internet gaming.<br>Patients with IGD were diagnosed according to the DSM-5 criteria and were also assessed using the Young's Internet addiction test (IAT).<br>Subjects with scores of at least 70 on the IAT who spent more than 4 h per day and 30 h per week using the Internet were included.	N/A	Increased regional homogeneity in the posterior cingulate cortex may be a common neurobiological feature of Internet Gaming Disorder, and alcohol use disorder.	-
10	King, Adair, Saunders, &	Australia	Identify variables predictive of short-term commitment to gaming abstinence	186<br>adult gamers with gaming-related	Internet gaming disorder (IGD; APA, 2013);	(1) meeting five or more DSM-5 IGD criteria and personal	Potential participants were individuals who visited a dedicated problem gaming	N/A	Abstainers were less likely to have withdrawal symptoms and less likely to play action	Selection Bias;

	Delfabbro (2018)		following initial voluntary contact with an online help service	problems were recruited online.	Internet Gaming Withdrawal Scale (IGWS; Flannery, Volpicelli, & Pettinati 1999); Internet Gaming Cognition Scale (IGCS; Forrest, King, & Delfabbro, 2017) Depression Anxiety Stress Scales–Short Form (DASS-21; Lovibond, & Lovibond, 1995); Eating Disorder Quality of Life Scale (EDQLS; Adair et al., 2007)	acknowledgement of gaming problems; (2) willingness to attempt to abstain from games for at least 7 days;	community support site that provides resources to quit or reduce gaming. The survey consisted of the following subjects: (1) current and historical use of games, (2) gaming preferences, (3) structural characteristics, (4) gaming-related cognitions, (5) craving and withdrawal, (6) disordered gaming, (7) mood symptoms, and (8) treatment-seeking Histories<br><br>A one-week follow up survey was administered. Participants was selected in according with inclusion criteria.		shooting games. Participants with mood symptoms reported significantly more Internet gaming disorder symptoms, stronger maladaptive gaming cognitions more previous occurrences of gaming problems, and poorer quality of life.	Attrition Bias;
11	Ko et al., (2014)	Taiwan	(1) evaluate the diagnostic validity of individual criteria of Internet gaming disorder in the DSM-5 and the criteria of craving and irritability; (2) determine the optimal cut-off point for the Internet gaming disorder criteria in the DSM-5.	75 subjects with Internet gaming disorder; 75 without Internet gaming disorder; 75 in remission	Diagnostic criteria of Internet addiction for college students (DC-IA-C; Ko et al., 2010); The diagnostic criteria of IGD in DSM-5 (American Psychiatric Association, 2013); Mini international neuropsychiatric interview (MINI; Sheehan et al., 1998); Questionnaire on gaming urge-brief version (QGU-B; Cox et al., 2001) ; Chen Internet Addiction Scale (CIAS ; Chen et al., 2003).	(1) game play time ≥ 4h per day or ≥ 8h per day on weekends or > 40h per week; (2) maintaining a pattern of Internet gaming for more than two years. (3) interview based on the DC-IA-C criteria specified for online gaming behaviour in this study Interview by a psychiatrist.	All participants were recruited by advertisement. Participants who met all inclusion criteria underwent a further interview by a psychiatrist based on the DC-IA-C to determine the diagnosis of Internet gaming disorder . The criteria were specific for online gaming behaviour Only functional impairment contributed by online gaming was recruited for diagnosis.	N/A	Except for the “deceiving” and “escape” criteria, all criteria of IGD had diagnostic accuracy ranging from 77.3% to 94.7% to differentiate university students with IGD from remitted students. Fulfilling 5 or more criteria of IGD in the DSM-5 was the best cut-off point to differentiate young adults with IGD from healthy or remitted users.	-
12	Lee et al., (2017)	South Korea	The purpose of the current study was to assess the temperament and character of subjects with Internet gaming disorder in comparison with those with alcohol dependence	83 patients with internet gaming disorder; 95 adults with high risk of alcohol dependence; 92 healthy comparison subjects.	Young Internet Addiction Scale (IAT; Young, 1996); Alcohol Use Disorder Identification Test (AUDIT; Bush et al., 1998) Structured Clinical Interview for DSM–IV (APA, 2000) Beck Depression Inventory II (BDI-II; Beck et al., 1996) Beck Anxiety Inventory (BAI; Beck et al., 1988); Adult ADHD Rating Scale (AADHD; Kim, 2003); Behavioural Activation System (BAS; Carver & White, 1994).	(1) game play time ≥ 4h per day and > 30h per week; (2) score > 50 on the IAT (3) Impaired behaviours or distress due to a maladaptive pattern of Internet gaming.	Adults who visited the Online Game Clinic at University Hospital with problematic Internet gaming and that meet inclusion criteria were included. Two psychiatrists evaluated and diagnosed all participants using the structured clinical interview for the DMS-IV (SCID-IV).	N/A	Among patients with IGD, the severity of Internet gaming disorder was positively correlated with novelty seeking score, impulsiveness and attention.	-

13	Mallorqui-Bague et al., (2017)	Spain	Comparing Internet gaming disorder with gambling disorder patients	A total of 288 adult patients (261 online gambling disorder and 27 Internet gaming disorder)	Yale Food Addiction Scale (YFAS-S; Gearhardt, Corbin, & Brownell, 2009); Symptom Checklist-90-R" (Derogatis, 1994) Temperament and Character Inventory – Revised" (Cloninger & Przybeck, 1994)	Semi-structured face-to-face clinical interview by experienced psychologists in according with the 9 proposed "DSM-5 criteria," (cut-off ≥ 5).	All participants were consequently referred through general practitioners or through another health care professional for problematic gambling or Internet gaming to the Bellvitge University Hospital Gambling Disorder Unit within the Department of Psychiatry. Experienced psychologists conducted two face-to face clinical interviews before a diagnosis was given. Only patients who sought treatment for online GD or IGD as their primary health concern were admitted	N/A	Internet gaming disorder and online gambling disorder patients share some emotional distress and personality traits.	-
14	Müller, Beutel, Egloff, & Wölfling, (2014)	Germany	Evaluate the relationships between personality traits and internet gaming disorder.	115 patients with internet gaming disorder.<br>167 control subjects	Scale for the Assessment of Internet and Computer Game Addiction (AICA-S; Wölfling, Müller, & Beutel, 2011) NEO Five-Factor Inventory (NEO-FFI; Costa, & McCrae, 1992) Berlin Inventory for Gambling (BIG; Grüsser, Hesselbarth, Albrecht, Mörsen, 2006)	AICA-S ≥ 13.5;<br>AICA-C ≥ 13<br><br>Exclusion criteria: major depression, bipolar disorder, psychotic disorder, substance use disorders, any personality disorder and pathological gambling (online or offline).	Participants consisted of treatment seekers from an outpatient clinic in Germany meeting criteria for IGD according to a self-report measure (Scale for the Assessment of Internet and Computer Game Addiction; AICA-S) and an external expert rating (Checklist for the Assessment of Internet and Computer Game Addiction; AICA-C)	N/A	Internet gaming disorder was associated with higher neuroticism, decreased conscientiousness and low extraversion.	-
15	Nam, Bae, Kim, Hong, & Han, (2017)	South Korea	Evaluation of bupropion and escitalopram on reducing the severity of depressive symptoms and internet gaming disorder symptoms in patients with both major depressive disorder and internet gaming disorder.	34 patients (15 bupropion group + 15 escitalopram group) with both problematic internet game play and Major Depression Disorder	Young Internet Addiction Scale (IAT; Young, 1996); Beck Depression Inventory II (BDI-II; Beck et al., 1996) Structured Clinical Interview for DSM–IV (APA, 2000) Korea Attention Deficit Hyperactivity Disorder Scale (K-ARS; KIM et al., 2003) Behavioural Inhibition and Activation Scales (BIS-BAS; Carver, & White, 1994).	Clinical Interview for the Diagnostic and Statistical Manual of Mental Disorders 4th edition (DSM-IV) and diagnosed by a psychiatrist<br><br>(1) game play time ≥ 4h per day or > 30h per week;<br>(2) score > 50 on the IAT<br>(3) impaired behaviours or distress due to a maladaptive pattern of Internet gaming.	All patients were screened with the structured Clinical Interview for the Diagnostic and Statistical Manual of Mental Disorders 4th edition (DSM-IV) and diagnosed by a psychiatrist. Problematic internet game play was defined by the inclusion criteria	Bupropion; Escitalopram;<br><br>The patients were started on bupropion sustained-release 150 mg/day and increased to 300 mg/day during the first week of treatment.<br>The patients were started on escitalopram 10 mg/day and increased to 20 mg/day during the first week of treatment. During weeks 2 to 12, the patients were asked to maintain a consistent medication dose.	Bupropion showed greater effects than escitalopram on reducing impulsivity and attentional symptoms.	-
16	Pallesen, Lorvik, Bu, & Molde, (2015)	Norway	Investigated the effects of a manualized therapy for video game addiction	12 males, ages 14–18 suffering from video game addiction	Game Addiction Scale for Adolescents (GASA; Lemmens, et al ., 2009). Problem Video Game Playing Scale (PVGPS; Salguero & Moran, 2002). The Clinical Global Impression scale (GCI; Guy, 1976)	(1) GASA ≥ 3;<br>and/or<br>(2) 4 or 5 on all three items of the PVGPS	Participants suffering from video game addiction, were recruited via newspaper advertisements. All scored 3 or higher on all the seven items of the short version of the Game Addiction Scale for Adolescents, and/or their mothers scored 4 or 5 on all three items of the parent version of the Problem Video Game Playing Scale.	Manualized therapy based on cognitive behavioural therapy, short-term strategic family therapy, solution-focused therapy, and motivational interviewing.<br><br>The therapy was conducted by psychologists with formal clinical specialty certification in working with addiction and dependency problems.	Patients reported moderate improvement Mothers reported statistically significant improvement.<br>The therapists reported marked treatment response in six of the 12 patients.	-

							Psychologist conducted the therapy. Diagnosis is not reported.	The sessions were given on a weekly basis in addition to home assignments.		
17	Park, Han, Kim, Cheong, & Lee, (2016a)	South Korea	Examine wherever the genre of the online game is associated with impulsivity and sociality in individuals with online game addictions	212 patients with problematic online game playing were divided into four groups by game genre: 1) massive multiplayer online role playing game, 2) real-time strategy, 3) first-person shooter, and 4) other	Young Internet Addiction Scale (IAT; Young, 1996);<br>Rosenberg Self-Esteem Scale (RSES; Rosenberg, 1965)<br>Behavioural Inhibitory System(BIS; Carver & White, 1994);<br>Behavioural Activation BAS; Carver & White, 1994).)<br>Beck Depression Inventory II (BDI-II; Beck et al., 1996)<br>Beck Anxiety Inventory (BAI; Beck et al., 1988);<br>Attention-Deficit/Hyperactivity Disorder (ADHD; Dupaul, 1991)<br>Family Environmental Scale (FES; Moos, & Mood, 2002)<br>Social Avoidance and Distress Scale (SADS; Watson, & Friend, 1969).<br>Wechsler Adult Intelligent Scale (WAIS; Wechsler, 1955)	(1) game play time ≥ 4h per day or > 30h per week;<br>(2) score > 50 on the IAT;<br>(3) irritable, anxious, and aggressive behaviours when forced to stop online game play;<br>(4) impaired behaviours or distress, economic problems, and maladaptive patterns in regular life due to excessive online game play;<br>(5) irregular life pattern due to disrupted diurnal rhythms, school truancy, or loss of job.	Patients with problematic online game play who visited the Online Game Clinic Center. All patients were screened using the structured clinical interview of DSM-IV for psychiatric comorbidities.<br>Participants that met inclusion criteria were included.	N/A	The genre of online game was not associated with impulsivity, but social anxiety status varied significantly with game genre, and differences in social anxiety were especially pronounced in patients playing the MMORPG. In addition, self-esteem was highest in the real-time strategy game genre.	-
18	Park, Kim, Kim, & Choi, (2017)	South Korea	To determine neurophysiological markers of P300 components associated with changes in symptoms after outpatient management with pharmacotherapy in patients with Internet gaming disorder.	47 male participants (18 with Internet gaming disorder and 29 healthy controls)	Young Internet Addiction Scale (IAT; Young, 1996);<br>Beck Depression Inventory II (BDI-II; Beck et al., 1996)<br>Beck Anxiety Inventory (BAI; Beck et al., 1988);<br>Wechsler Adult Intelligent Scale (WAIS; Wechsler, 1955)<br>Structured Clinical Interview for DSM–IV (APA, 2000)<br>EEG recording<br>ERP analysis	Diagnosis of Experienced psychiatrist based on the criteria of the DSM-5.<br>IAT score criteria (Cut-OFF not reported).	All patients had sought treatment at an outpatient clinic of Boramae Medical Center, Seoul, South Korea, due to excessive participation in Internet gaming, and IGD was diagnosed by an experienced psychiatrist based on the criteria of the DSM-5.	6-month outpatient management program that included pharmacotherapy with a selective serotonin reuptake inhibitor using the following average daily doses: escitalopram at 15.83 ± 9.17 mg, fluoxetine at 50.00 ± 9.17 mg, or paroxetine at 30.00 ± 14.14 mg.	Reduced P300 amplitudes and delayed latencies are candidate endophenotypes in the pathophysiology of IGD.	Reporting Bias

19	Park et al., (2016b)	South Korea	Verify that virtual reality therapy for online gaming addiction would improve the functional connectivity of the cortico-striatal-limbic circuit by stimulating the limbic system.	Twenty-four adults with online gaming addiction. 12 in the cognitive behaviour therapy group and 12 in the virtual reality therapy group.	Young Internet Addiction Scale (IAT; Young, 1996);<br>Beck Depression Inventory II (BDI-II; Beck et al., 1996)<br>Beck Anxiety Inventory (BAI; Beck et al., 1988);<br>Attention-Deficit/Hyperactivity Disorder (ADHD; Kessler, et al., 2005)<br>Structured Clinical Interview for DSM–IV-TR (APA, 2000)<br>fMRI assessment	(1) game play time > 30h per week;<br>(2) score > 50 on the IAT;<br>(3) disruption of regular life due to excessive Internet gaming;<br>(4) maladaptive behaviours or distress in school or work due to excessive Internet gaming;	Through advertisements posted at Chung-Ang University Medical Center were recruited.<br>All participants were screened by a trained psychiatrist based on the Structured Clinical Interview for DSM-IV-TR Axis-I-Disorders, Patient-Edition (SCID-I/P).<br>All participants were evaluated through an interview regarding excessive game playing patterns and completed the 20-item Young's Internet Addiction Scale	Cognitive behaviour therapy (CBT)<br>Eight-session CBT by a group of experts including a psychiatrist, nurse, psychologist, and social worker.<br>An expert led each session associated with their area of knowledge.<br>Each session lasted for about 2 h.<br><br>Virtual reality therapy (VRT)<br>Eight-session VRT, which consisted of three steps.<br>A trained psychiatrist carried out all treatment procedures by instructing the participant and managing the VR system using a keyboard and microphone in the control room.	VRT seemed to reduce the severity of OGA, showing effects similar to CBT, and enhanced the balance of the cortico-striatal-limbic circuit.	Reporting Bias
20	Sakuma et al., (2017)	Japan	Investigate the efficacy of the Self-Discovery Camp (SDiC) and the correlations between individual characteristics and outcome measures.	10 patients with Internet gaming disorder	Stages of Change Readiness and Treatment Eagerness Scale (SOCRATES; Miller, & Tonigan, 1996)	1) satisfied both Griffith's six components of addiction and the DSM-5 IGD criteria through an interview by one our center's psychiatrists	Participants were diagnosed with IGD according to DSM-5 criteria. They also fulfilled Griffith's six components of addiction: salience, mood modification, tolerance, withdrawal, conflict, and relapse (Griffiths, 2005);<br><br>All participants were recruited from the center's population of inpatients and outpatients with IGD. They were all diagnosed with IGD by psychiatrists prior to being recruited.	The Self-Discovery Camp (SDiC), a Japanese version of a therapeutic residential camp. It is a camp that was held over 8 nights and 9 days in the National Central Children's Center, Gotenba City, Japan.<br><br>All participants stayed in the dormitory for the duration of the program. They were not allowed to bring PCs, mobile phones, gaming machines, or any other digital equipment.<br>The SDiC comprised 14 sessions of CBT with clinical psychologists, 3 medical lectures provided by doctors, 8 sessions of personal counseling, and a workshop entitled, "Our relationship with the Internet."	Total gaming time was significantly lower 3 months after the SDiC. Problem recognition and self-efficacy towards positive change also improved	-
21	Torres-Rodríguez, Griffiths, Carbonell, Farriols-Hernando, & Torres-Jimenez, (2017)	Spanish	Explore efficacy of the PIPATIC program for adolescents with Internet Gaming Disorder	4 male adolescents aged between 13 and 18 years	Video Game-Related Experiences Questionnaire (CERV; Chamarro et al. 2014);<br>Internet Gaming Disorder Test (IGD-20 Test; Pontes et al. 2014)<br>Symptom Checklist-90-R (SCL-90-R; Derogatis 1996)<br>Youth Self-Report for ages 11–18 years (YSR/11–18; Achenbach, & Rescorla, 2001)	1) Endorsing at least five or more of the nine IGD criteria according to DSM-5 (APA 2013),<br>2) scoring 71 or more on IGD-20 Test (Pontes et al. 2014)<br>3) CERV ≥ 39<br>4) MACI ≥ 60	adolescents diagnosed with IGD at two public mental health centers in the Barcelona metropolitan area by clinical psychologists.	PIPATIC program (Programa Individualizado Psicoterapéutico para la Adicción a las Tecnologías de la Información y la Comunicación)<br>The program comprises six modules:<br>1) Psychoeducational module.<br>2) Standard IGD intervention module.<br>3) Intrapersonal module. 4) Interpersonal module.	After completing the PIPATIC program, all participants showed clinical improvement in the amount of time spent using video games and in the symptoms of IGD.	-

					Family Environment Scale (FES; Moos and Moos 1994) Working Alliance Theory of Change Inventory (WATOCI; Horvath and Greenberg 1986)			5) Family module. 6) Development of a new lifestyle module.		
22	Van Rooij, Schoenmakers, & Van De Mheen, (2016)	Netherlands	Explore the Clinical validation of the C-VAT 2.0	32 clinical youth sample in treatment for video gaming disorder.	Clinical Video game Addiction Test (C-VAT 2.0; Van Rooij, et al., 2016) Video game Addiction Test (VAT; Van Rooij, Schoenmakers, et al., 2012) Global Assessment of Functioning (Havenaar, Van Os, & Wiersma, 2004)	Use of clinical professional opinion of the treating therapist as a main point of reference (Tao et al., 2010). Interview with standardized set of questionnaires of Dutch addiction care	Patients were only included in the study if they reported directly for gaming disorder, or if gaming related activities were readily apparent in the intake session. The study involved only youth clinics for addiction care. Patients who report for treatment in Dutch addiction care are generally interviewed using a standardized set of questionnaires about addictions during intake. For the current study, existing instruments were supplemented with the C-VAT 2.0.<br><br>Participants reported spending pretty much all their free time and even part of their school time on gaming;<br>problems with their social environment;<br>Decline in school performance	N/A	C-VAT 2.0 shows preliminary validity in a sample of gamers in treatment for gaming disorder, but the discriminating value of the instrument should be studied further.	Attrition bias
23	Vasiliu & Vasile, (2017)	N/R	Examine efficacy of cognitive-behavioural therapy in a case of Internet gaming disorder and alcohol-use	Single Case - Male patient 24-year old.	Internet Gaming Disorder Scale- Short Form (IGDS-SF; Sarda, Begue, Bry, Gentile, 2016) Alcohol Use Disorders Identification Test (AUDIT; Bush et al., 1998) Global Assessment of Functioning Scale (GAF: APA, 2000)	1) on-line gaming ≥ 7 hours spent daily<br>2) negative academic consequences<br>3) onset 18 months ago<br>4) gradually increase of hours spent daily in gaming activities.	A Psychiatry conduct the diagnosy. Significant financial losses;<br>Conflicts in relationships because of gaming;<br>Decline in academic performance<br>Increasing time of gaming.<br>Life problems because of gaming	Cognitive- behavioural therapy (CBT) oriented techniques were applied during the 10 sessions of therapy, with bi-weekly sessions, using mainly cognitive restructuring with a daily register of automatic thoughts that triggered problematic behaviours.<br><br>Thoughts-emotions-behaviours;<br>Coping skills techniques;<br>Relaxation techniques ;<br>Attention-switch methods and daily programming of activities.	The therapy reduced time spent on Internet gaming and alcohol use.	Reporting Bias
24	Yao et al., (2017)	China	Evaluate the efficacy of a group behavioural intervention combining reality therapy and mindfulness meditation in reducing decisional impulsivity and Internet gaming Disorder severity.	25 Internet gaming Disorder and 21 healthy comparison	Chen Internet Addiction Scale (CIAS; Chen, Weng, Su, Wu, & Yang, 2003) Beck Depression Inventory II (BDI-II; Beck et al., 1996) Beck Anxiety Inventory (BAI; Beck et al., 1988);	Interview on DSM-5 criteria of IGD (American Psychiatric Association, 2013). Inclusion Criteria:<br>(1) met 5 or more criteria DSM-5<br>(2) spent at least 14 h per week on Internet gaming	Participants were recruited by means of online advertisements and word of mouth. Participants were interviewed using DSM-5 criteria of IGD and and those who met inclusion criteria were diagnosed as individuals with IGD.	6-week group behavioural intervention combining reality therapy and mindfulness meditation and repeated the test one week after the end of the intervention.<br><br>The group behavioural intervention was conducted weekly, including six 2-h sessions, with 9-10 individuals	Internet gaming Disorder subjects were decreased in delay discounting rate and Internet gaming Disorder severity	-

					Decisional impulsivity tasks (DDT, Kirby, Petry, & Bickel, 1999<br>Decision Making (BART; Lejuez et al., 2002)	(3) reported Internet games as their primary Internet.		with IGD in each group talking of specific topics of IGD based on the framework of reality therapy.<br>Each topic and related activity would last for approximately 110 min.<br>A brief mindfulness meditation was conducted in the remaining 10 min to end each session.		
25	Yeh et al., (2017)	Taiwan	Evaluates the association between procrastination and Internet gaming Disorder	87 individuals with Internet gaming Disorder and 87 Controls.	The DSM-5 diagnostic criteria for IGD (American Psychiatric Association, 2013);<br>Modules of psychotic disorders (MINI; Sheehan et al., 1998)<br>Procrastination scale (PS; Lay, 1986);<br>Clinical global impressions scale (CGI; Busner and Targum, 2007);<br>Center for Epidemiological Studies' Depression Scale (CES-D; Radloff, 1977);<br>Buss–Durkee Hostility Inventory-Chinese Version-Short Form (BDHIC-SF; Lin et al., 2008);<br>Barratt Impulsiveness Scale (BIS-11; Barratt, 1985).	Interview on the basis of the DSM-5 diagnostic criteria for IGD (American Psychiatric Association, 2013).<br>1) Play internet games ≥ 4h per day on weekdays and ≥ 8h per day on weekends or ≥ 40h per week<br>2) Internet gaming pattern ≥ 2 years.	A semi-structured interview to examine the DSM-5 criteria for IGD The DSM-5 diagnostic criteria for IGD comprise nine items:<br>preoccupation, withdrawal, tolerance,<br>unsuccessful attempts to control,<br>loss of other interests,<br>continued excessive use despite psychosocial problems,<br>deceiving others regarding use,<br>escapism, and functional impairment<br>Participants fulfilling five or more criteria were included in the IGD group.	N/A	Procrastination is associated with Internet gaming Disorder independent of depression, hostility, and impulsivity.	-
26	Zhang et al., (2018)	China	Investigate how the Cortical-ventral striatum circuitry responds to psychobehavioural interventions in internet gaming disorder	74 internet gaming disorder subjects and 41 healthy controls	Functional magnetic resonance imaging (fMRI)<br>Chinese Internet addiction scale (CIAS; Chen et al. 2003)	1) CIAS > 67<br>2) more than half of the time spent online was on games;<br>3) Time spent on Internet gaming per week ≥14h (with Internet gaming ≥ 2h every day)	college students were screened through<br>online questionnaires and telephone. Students that met inclusion criteria was selected.	The Craving behavioural intervention (CBI) is a behavioural intervention program developed to reduce craving for Internet gaming, based on theories of the craving framework of boundary conditions and the fulfilment of psychological needs for Internet use.<br><br>Subjects receive six sessions of CBI, conducted weekly with each session lasting for 2.5 hours.<br>In addition, mindfulness training was administrated for about 20 minutes at the end of each session	Role of the Cortical-ventral striatum circuitry to left inferior parietal lobule, and of the right inferior frontal gyrus and left middle frontal gyrus, in the effective treatment of Internet addiction.	Selection Bias;<br>Attrition bias
27	Zhang et al., (2016a)	China	Evaluate the benefits of interventions for internet gaming disorder is	36 young adults with internet gaming disorder and 19 healthy comparison subjects.	fMRI scanning;<br>Chinese Internet addiction scale (CIAS; Chen et al. 2003);	1) CIAS > 67;<br>2) game play time > 20h per week for a minimum of one year;	Participants were recruited by means of online advertisements and word of mouth and were selected through an online questionnaire and telephone	The Craving behavioural intervention (CBI) was developed on the basis of behavioural intervention, the craving framework of	Internet gaming disorder subjects showed decreased amplitude of low fluctuation in the orbital frontal cortex and posterior cingulate cortex.	Selection Bias;<br>Attrition bias

					Fagerstrom Test for Nicotine Dependence (FTQ; Fagerström, 1978);<br>Alcohol Use Disorders Identification Test (AUDIT; Bush et al., 1998);<br>Beck Depression Inventory II (BDI-II; Beck et al., 1996)<br>Beck Anxiety Inventory (BAI; Beck et al., 1988);	3) Internet gaming as their primary online activity	screening. Participants were recruited according to inclusion criteria.	boundary conditions, and the fulfillment of psychological needs for Internet use.<br><br>The group intervention was conducted once a week, including six 2.5-hour sessions, with 8–9 IGD subjects in each group.		
28	Zhang et al., (2016b)	China	Examine the effects of a craving behavioural intervention (CBI), on cue-induced craving and neural activation in regions involved in reward and motivational processing.	44 Internet gaming disorder subjects and 22 healthy control	fMRI scanning;<br>Chinese Internet addiction scale (CIAS; Chen et al. 2003);<br>Fagerstrom Test for Nicotine Dependence (FTQ; Fagerström, 1978);<br>Alcohol Use Disorders Identification Test (AUDIT; Bush et al., 1998);<br>Beck Depression Inventory II (BDI-II; Beck et al., 1996)<br>Beck Anxiety Inventory (BAI; Beck et al., 1988);	1) CIAS ≥ 67;<br>2) game play time > 14h per week for a minimum of one year;<br>3) One of the most popular gaming as their primary online activity;	Participants were recruited by means of online advertisements and word of mouth and were selected through an online questionnaire and in-person semistructured screening. Participants were recruited according to inclusion criteria.	The Craving Behavioural Intervention (CBI) was developed on the basis of behavioural intervention, the craving framework of boundary conditions, and the fulfillment of psychological needs for Internet use.<br><br>The group intervention was conducted once a week, including six 2.5-hour sessions, with 8–9 IGD subjects in each group.	The CBI group showed decreased internet gaming disorder severity and cue-induced craving, enhanced activation in the anterior insula and decreased insular connectivity with the lingual gyrus and precuneus after receiving CBI.	Selection Bias; Attrition bias

Note: N/A = Not Applicable; N/R = Not Reported.
